# Variability in contact precautions to control the nosocomial spread of multi-drug resistant organisms in the endemic setting: a multinational cross-sectional survey

**DOI:** 10.1186/s13756-018-0366-5

**Published:** 2018-07-09

**Authors:** Danielle Vuichard Gysin, Barry Cookson, Henri Saenz, Markus Dettenkofer, Andreas F. Widmer, for the ESCMID Study Group for Nosocomial Infections (ESGNI)

**Affiliations:** 1 Department of Infectious Diseases and Hospital Epidemiology, University Hospital Basel, 4051 Basel, Switzerland; 2 Division of Infection and Immunity, University College London, London, UK; 3 ESCMID Executive Office, Basel, Switzerland; 4 Institute of Hospital Hygiene and Infection Prevention, Gesundheitsverbund Landkreis Konstanz, Radolfzell, Germany; 5 Present address: Department of Internal Medicine, Cantonal Hospital Thurgau, Muensterlingen, Switzerland

**Keywords:** Contact precaution, Isolation, Multi-drug resistant organisms, Implementation, Barriers

## Abstract

**Background:**

Definitions and practices regarding use of contact precautions and isolation to prevent the spread of gram-positive and gram-negative multidrug-resistant organisms (MDRO) are not uniform.

**Methods:**

We conducted an on-site survey during the European Congress on Clinical Microbiology and Infectious Diseases 2014 to assess specific details on contact precaution and implementation barriers.

**Results:**

Attendants from 32 European (EU) and 24 non-EU countries participated (*n* = 213). In EU-respondents adherence to contact precautions and isolation was high for Methicillin-resistant *Staphylococcus aureus* (MRSA), carbapenem-resistant Enterobacteriaceae, and MDR *A. baumannii* (84.7, 85.7, and 80%, respectively) whereas only 68% of EU-respondents considered any contact precaution measures for extended-spectrum-beta-lactamase (ESBL) producing non-*E. coli*. Between 30 and 45% of all EU and non-EU respondents did not require health-care workers (HCW) to wear gowns and gloves at all times when entering the room of a patient in contact isolation. Between 10 and 20% of respondents did not consider any rooming specifications or isolation for gram-positive MDRO and up to 30% of respondents abstain from such interventions in gram-negative MDRO, especially non-*E. coli* ESBL. Understaffing and lack of sufficient isolation rooms were the most commonly encountered barriers amongst EU and non-EU respondents.

**Conclusion:**

The effectiveness of contact precautions and isolation is difficult to assess due to great variation in components of the specific measures and mixed levels of implementation. The lack of uniform positive effects of contact isolation to prevent transmission may be explained by the variability of interpretation of this term. Indications for contact isolation require a global definition and further sound studies.

**Electronic supplementary material:**

The online version of this article (10.1186/s13756-018-0366-5) contains supplementary material, which is available to authorized users.

## Background

The European Society of Clinical Microbiology and Infectious Diseases (ESCMID) and Healthcare Infection Control Practices Advisory Committee (HICPAC) have defined multidrug-resistant organisms (MDRO) that qualify for contact precautions and isolation [1]. According to HICPAC, contact precaution measures are indicated if transmission of an infectious agent is not interrupted by the use of standard precautions alone due to environmental contamination and, therefore, requires HCW to wear gloves and gowns upon room entry, not only if contact with blood or body fluid is anticipated. HICPAC also recommends that such patients should be placed preferably in a single room [2]. Similarly, the guidelines on prevention of transmission of gram-negative MDRO issued by ESCMID require contact precaution for patients colonized or infected with an epidemiologically targeted organism, that includes wearing gloves and gowns upon entry to the room and the use of patient-dedicated or single-use disposable non-critical equipment [3] (Table 1).

**Table 1:** Core elements of contact precautions (CP) recommended by recent ESCMID and HICPAC/CDC guidelines

	ESCMID 2014 (3)	HICPAC/CDC 2007 (2)
Indication for CP	Colonization or infection with MDRO	Colonization or infection with MDRO
Donning and wearing of gloves and gowns	Gown and gloves are donned upon entry to a room	Gown and gloves are donned upon room entry Gown and gloves are indicated for all interactions that may involve contact with the patient or potentially contaminated areas in the patient’s environment.
Disposal of gowns and gloves	Not stated	Gown and gloves are discarded before exiting the patient room
Additional requirements & recommendations	Use of disposable single-use or patient-dedicated non-critical care equipment (e.g. blood pressure cuffs and stethoscopes).	Use of patient-dedicated or single-use disposable noncritical equipment
Placement of patients	Special isolation wards Nursing cohort with separate rooms on general wards Single room or cohort in same room without dedicated personnel Placement in a room with patients unaffected by MDROs but maintaining CP by use of gowns and gloves based on the patient’s extent of MDRO carriage	Single patient room preferred Cohort patients with same MDRO Multi-bed rooms with non-infected/non-colonized patients: at least 3 ft spatial separation between beds

*CDC* centers for disease control and prevention, *CP* contact precaution, *ESCMID* european society of clinical microbiology and infectious diseases, *HICPAC* healthcare infection control practices advisory committee, *MDRO* multidrug resistant organism

However, there is no uniform definition of multidrug resistance in gram-negative bacteria and the indications to implement isolation precaution measures for MDRO vary substantially [4]. Reasons for this are not well understood. The variability in practices and the strictness of implementation (e.g. whether gowns and gloves are worn upon room entry or only if contact with blood or bodily fluid is anticipated, or whether implementation of contact precaution and isolation depends on the presence or absence of patient risk factors), has not been well studied amongst health-care professionals. This is of major relevance when examining the success of prevention and control of the spread of MDRO and when designing studies to look at the effectiveness of such interventions. Interestingly, comparison of national MRSA guidelines of 13 European (EU) countries has also shown divergent implementation regarding donning of gloves and gowns [5].

## Methods

Our main survey aims were to explore the diversity in adopting contact precaution and isolation practices for gram-positive and gram-negative MDRO and to assess barriers to their implementation.

After an in-depth discussion amongst the ESCMID nosocomial infection study group (ESGNI) committee members we decided to focus on the indication, circumstances and implementation of contact precautions and isolation for MRSA, glycopeptide-resistant enterococci (GRE), extended-spectrum-beta-lactamase-producing Enterobacteriaceae (ESBL-E) and carbapenem-resistant Enterobacteriaceae (CRE), MDR *P. aeruginosa*, and MDR *A. baumannii*.

A questionnaire survey was developed by the authors and distributed amongst the ESGNI committee members for revision. Levels of agreement on barriers frequently encountered during implementation were measured on a 5-point Likert scale (3 being neutrality) [6]. The survey was then transferred onto Survey Monkey® [7] and pilot-tested among a broader group including five infection control nurses and five infectious diseases physicians from Switzerland, Germany and the USA.

The online survey was applied to attendees of the 2014 ECCMID in Barcelona, Spain. On-site participant recruitment was by study team members during the regular opening hours. Individuals were invited to complete the survey at a booth. Study team members addressed any issues of comprehension. As a recruitment incentive, there was a lottery with three prizes. In addition, the online survey was open to all ESCMID members for six weeks after the congress.

### Statistical analyses

Numbers, percentages, median and interquartile range (IQR) were used for descriptive statistics. Countries were categorised into EU and non-EU in compliance with a reference classification system [8]. We regrouped the transcontinental Eurasian countries, e.g. Turkey, as belonging to the Southern EU Area rather than Western Asia to be consistent with other publications [9]. We compared differences in proportions among EU and non-EU responders using Chi-square or Fisher’s exact test. Missing answers were removed from the respective analysis on a case-by-case basis. In our primary analysis, we considered all non-missing responses equivalently without taking potential nesting into account. In order to evaluate a possible overestimation of effects due to nested data, we eliminated all duplicates that we defined as respondents from the same country and from the same hospital size. We then repeated the primary analysis with the de-duplicated dataset. All analyses were performed using SPSS statistical software version 23.0 [10].

## Results

Overall, 213 individuals from 32 EU and 28 non-EU countries participated in the survey.The majority were European and had their workplace in either the Southern European Area (31%), in Western (22%), Northern (16%), or Eastern Europe (7%); about a quarter of the respondents came from countries out-side Europe (Asia and The Middle East 11%, South America 8%, Africa 5%). A total of 77 (36.1%) respondents were specialized in infection control and prevention and 108 respondents (50.7%) had either a background in microbiology and/or infectious diseases. The median experience in infection control was 8 (IQR: 3–15) years. There were 159 (74.6%) medical doctors and the majority (71.8%) worked in acute care. Details on the participants’ country of workplace and professional responsibilities are listed in the supplementary appendix. Numbers (%) of completely missing answers to questions concerning indications of contact precautions were: 14 (6.6) for MRSA, 38 (17.8) for GRE, 27 (12.7) for *E. coli* ESBL, 14 (6.6) for non-*E. coli* ESBL, 14 (6.6) for CR *E. coli*, 17 (8.0) for CRE (other than *E. coli*), 14 (6.6) for MDR *P. aeruginosa*, and 14 (6.6) for MDR *A. baumannii*. The proportion of EU-respondents reporting any form of contact precautions/isolation, irrespective whether a patient was colonized or infected, was high (≥ 80%) for MRSA, CRE (other than *E. coli*), and MDR *A. baumannii* (84.7, 85.7, and 80%, respectively) with lower, but still similar percentages among non-EU respondents (Table 2). The proportions amongst EU-respondents who would apply any form of contact precaution were markedly lower for ESBL-producing *E. coli* and non-*E. coli* ESBL (59.4 and 68%, respectively). Answers from EU and non-EU responders differed significantly regarding overall contact precaution indications for ESBL-E other than *E. coli* (*p* = 0.044) in that approximately one third of non-EU responders either did not consider any contact precaution measures or did not determine the presence of ESBL. Amongst those who implemented contact precautions more non-EU responders than EU-responders did so if the patient was only colonized (Table 2).

**Table 2:** Indication and specification for contact precautions (CP) and isolation^a^

	MRSA		GRE			*E. coli* ESBL			Non-*E. coli* ESBL			
	EU	Non EU	*p*-value^b^	EU	Non EU	*p*-value^b^	EU	Non EU	*p*-value^b^	EU	Non EU	*p*-value^b^
No CP	12.7	24.5	0.165	30.0	32.7	0.396	32.7	34.7	0.636	23.3	34.7	*0.044*
CP only if infected	16.7	10.2		54.7	44.9		14.7	10.2		17.3	6.1	
CP if colonised and/or infected	68.0	65.3		15.3	22.4		44.7	40.8		50.7	40.8	
Unknown	42.7	0		0	0		5.3	10.2		6.0	14.3	
ESBL not determined	n.a.	n.a.		n.a.	n.a.		2.7	4.1		2.7	33.3	
Total no. responses (%)	150 (75.4)	49 (24.6)		131 (74.9)	44 (24.1)		150 (75.4)	47 (24.6)		150 (75.4)	49 (24.6)	
Gowns and gloves whenever entering the room	57.3	62.9	0.398	59.0	56.7	0.623	44.9	59.1	0.234	47.3	71.4	*0.046*
Gowns and gloves if direct contact is anticipated	37.9	37.1		37.1	43.3		55.1	40.9		52.7	28.6	
Other procedures (e.g. standard precautions only)	4.8	0.0		3.8	0.0		0	0		0	0	
Total no. responses (%)	124 (78.0)	35 (22.0)		105 (77.8)	30 (22.2)		89 (80.2)	22 (19.8)		93 (81.6)	21 (18.4)	
Single room	62.5	63.8		59.4	56.3	0.703	31.4	31.3	0.960	36.4	27.1	0.494
Cohorting	17.4	10.6		16.1	16.7		19.3	18.		20.7	18.8	
Spatial separation^c^	10.4	6.4	0.235	9.8	6.3		13.6	16.7		13.6	20.8	
No specific measures	9.7	19.1		14.7	20.8		35.7	33.3		29.3	33.3	
Total no. responses (%)	144 (75.4)	47 (24.6)		143 (74.9)	48 (25.1)		140 (74.5)	48 (25.5)		140 (74.5)	48 (25.5)	
	Carbapenem resistant *E. coli*			Carbapenem resistant non-*E. coli*			MDR *P. aeruginosa*			MDR *A. baumannii*		
	EU	Non EU	*p*-value^b^	EU	Non EU	*p*-value^b^	EU	Non EU	*p*-value^b^	EU	Non EU	*p*-value^b^
No CP	11.3	16.3	0.745	8.2	18.4	0.246	13.3	20.4	0.597	9.3	20.4	0.171
CP only if infected	14.0	10.2		13.6	10.2		17.3	14.3		17.3	20.4	
CP if colonised and/or infected	65.3	63.3		72.1	65.3		60.0	59.2		62.7	51.0	
Unknown	9.3	10.2		6.1	6.1		9.3	6.1		10.7	8.2)	
Total no. responses (%)	150 (75.4)	49 (24.6)		147 (75.0)	49 (25.0)		150 (75.4)	49 (24.6)		150 (75.4)	49 (24.6)	
Gowns and gloves whenever entering the room	61.3	68.8	0.440	63.5	60.6	0.763	56.3	64.7	0.389	58.7	61.3	0.797
Gowns and gloves if direct contact is anticipated	38.7	31.3		36.5	39.4		43.7	35.3		41.3	38.7	
Total no. responses (%)	111 (77.6)	32 (22.4)		115 (77.7)	33 (22.3)		103 (75.2)	34 (24.8)		109 (77.9)	31 (22.1)	
Single room	64.1	41.7	*0.029*	71.6	47.9	*0.026*	56.7	43.8	0.156	61.6	45.8	0.067
Cohorting	13.4)	14.6		12.1	18.8		18.7	14.6		18.1	14.6	
Spatial separation^c^	9.9	20.8		7.1	14.6		12.7	18.8		8.7	18.8	
No specific measures	12.7	22.9		9.2	18.8		11.9	22.9		11.6	20.8	
Total no. responses (%)	142 (74.7)	48 (25.3)		141 (74.6)	48 (25.4)		134 (73.6)	48 (26.4)		138 (74.2)	48 (25.8)	

^a^Values are percentages (related to the corresponding total respondents) unless indicated otherwise

^b^A two-sided *p*-value of < 0.05 was considered statistically significant

^c^Shared room with MDRO-negative patients but with optical barrier (e.g. red margin on the floor) or separated by screen/curtains

The majority (> 56%) of EU responders reported donning of gowns and gloves upon entry into the room *at all times* for all MDRO except ESBL-E. However, only non-EU responders followed this practice in ESBL-E in contrast to EU responders, where a majority (55 and 53%, for *E. coli* and non-*E. coli*, respectively) indicated that donning of gowns and gloves was required only when patient-care was likely*.* The differences in proportions of EU and non-EU responders were statistically significant for ESBL-E other than *E. coli* (*p* = 0.046) (Table 2)*.* After removing potential duplicate answers, the discrepancy became even more evident with statistically significant lower proportions of responders from EU countries that had strict gowning and gloving at all times implemented for ESBL-producing *E. coli* (*p* = 0.017) and other Enterobacteriaceae (*p* = 0.005) (Additional file 1).

A majority of EU and non-EU country participants preferred single room placement for MRSA (62.5 and 63.8%) and GRE (59.4 and 56.3%) (Table 2). The answers, however, were less consistent for gram-negative MDRO. Only about one third of EU and non-EU responders advocated single room placement for ESBL-*E. major* differences between responses from participants from EU and non-EU countries were encountered for rooming specifications in CR *E. coli* and CRE (other than *E. coli*), where EU responders compared to non-EU responders favoured single room placement (64.1% vs. 41.7 and 71.6% vs. 47.9%, respectively) over cohorting or spatial separation, whereas responses from non-EU participants were more divergent among the different placement options. Differences in placements of patients with MDRO among EU and non-EU responders, however, were not statistically significant in the sensitivity analysis (see Additional file 1).

The answers were highly consistent among all participants and for any MDRO, except for MRSA, that pre-emptive contact precautions/isolation had a significant value, whereas only a minority considered limiting implementation of contact precautions to patients with certain risk factors (e.g. diarrhoea or urinary incontinence) in their local practice (Table 3). None of the differences between responses from EU and non-EU countries were significant after deduplication (Additional file 1). When comparing the responses between infection control practitioners (ICP) and non-ICPs, as well as the responses between clinicians and non-clinicians, we also detected significantly different approaches to infection control measures across different pathogens (Additional file 1: Tables S3-S10). However, the results also demonstrated large incongruities amongst ICPs as well as amongst clinicians as to what strictness level of contact precaution is pursued.

**Table 3:** Other specific requirements and conditions for contact precaution (CP)

MDRO	Origin of responses	Total responses	Additional pre-emptive CP based on patient’s history^a^	CP only required if specific risk factors present^b^	Additional pre-emptive CP but only if specific risk factors	None applicable	*p*-value^c^
MRSA	EU	126	117 (92.9)	1 (0.8)	1 (0.8)	7 (5.6)	*0.038*
	Non EU	38	30 (78.9)	1 (2.6)	2 (5.3)	5 (13.2)	
GRE	EU	94	81 (86.2)	3 (3.2)	1 (1.1)	9 (9.6)	0.265
	Non EU	30	24 (80.00)	2 (6.7)	2 (6.7)	2 (6.7)	
ESBL *E. coli*	EU	91	74 (81.3)	8 (8.8)	4 (4.7)	5 (4.7)	0.812
	Non EU	26	22 (84.6)	1 (3.8)	2 (7.7)	1 (3.8)	
ESBL non-*E. coli*	EU	101	86 (85.1)	5 (5.0)	3 (3.0)	7 (6.9)	0.131
	Non EU	23	18 (78.3)	3 (13.0)	2 (8.7)	0 (0.0)	
Carbapenem resistant *E. coli*	EU	120	103 (85.8)	4 (3.3)	4 (3.3)	9 (7.5)	0.465
	Non EU	36	33 (91.7)	0 (0.0)	2 (5.6)	1 (2.8)	
Carbapenem resistant Enterobacteriaceae (non-*E. coli*)	EU	126	111 (88.1)	2 (1.6)	4 (3.2)	9 (7.1)	0.560
	Non EU	37	33 (89.2)	1 (2.7)	2 (5.4)	1 (2.7)	
MDR *P. aeruginosa*	EU	112	96 (85.7)	3 (2.7)	4 (3.6)	9 (8.0)	0.178
	Non EU	35	31 (88.6)	1 (2.9)	3 (8.6)	0 (0.0)	
MDR A. baumannii	EU	120	107 (89.2)	1 (0.8)	5 (4.2)	7 (5.8)	0.758
	Non EU	33	31 (91.2)	0 (0.0)	2 (5.9)	1 (2.9)	

^a^Formerly positive for respective MDRO or presumptive infection/colonization with respective MDRO

^b^CP only when certain risk factors present e.g. incontinence, diarrhoea, draining wounds

^c^A two-sided p-value of < 0.05 was considered statistically significant

Most respondents demonstrating poor knowledge were either no medical doctors, were not working in hospitals or had fewer years of experience in infection control (Additional file 1 Table S13).

### Most commonly encountered barriers

**Fig. 1:**
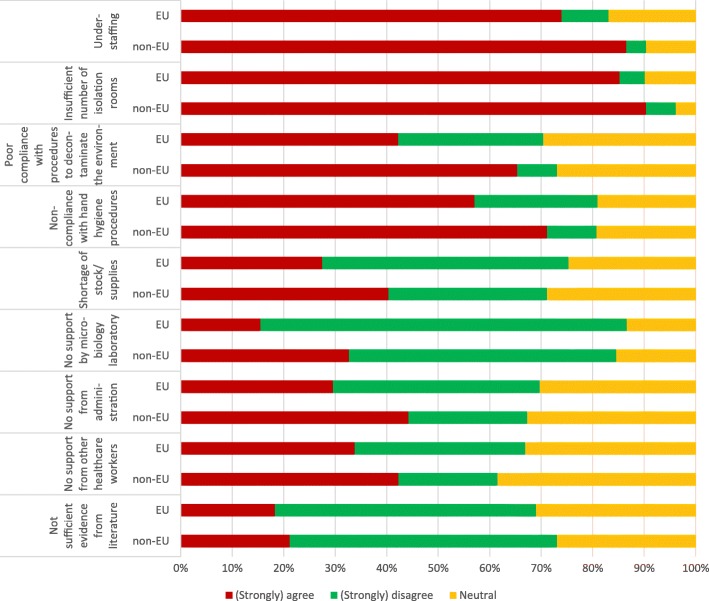
Most commonly encountered barriers when trying to implement contact precaution and isolation measures from the survey respondents’ perspective (*n* = 194, 4 missing from non-EU and 15 missing from EU countries)

Of the 213 participants, 15 (10%) Europeans and 4 (7%) non-Europeans did not respond to these questions. Respondents from EU- and non-EU countries largely agreed that the major obstacles to implement appropriate contact precaution/isolation measures were shortage of personnel (EU-respondents: 67%; non-EU respondents: 80%) and lack of rooms for isolation (77 and 84%, respectively). The opinions were more divergent between EU- and non-EU-respondents regarding lack of environmental cleanliness (EU-respondents: 38%, non-EU respondents: 61%), support from administration (27 and 41%, respectively) or microbiology (14 and 30%, respectively), and provision of supplies (25 and 38%, respectively), where non-EU respondents perceived more frequent constraints than EU respondents (Fig. 1).

## Discussion

MDRO comprise a global threat [11] causing economic damage comparable to the 2008 financial crisis [12]. International experts rated their control the highest priority [13]. Surprisingly, to the best of our knowledge, this is the first multinational survey addressing specifically potential differences and major hindrances in practical implementation of contact precaution/isolation measures in MDROs. Representatives from most European countries and from a large number of non-EU countries across Africa, Asia, and South America participated. The results have confirmed our suspicions that indications and practical implementation of contact precautions including isolation measures vary considerably. This study also showed there were major inconsistencies particularly in the handling of ESBL-E, CR *E. coli*, and CREs.

Firstly, in contrast to ESCMID [3] recommendations, 23.3% of EU-respondents did not consider any contact precaution measures in non-*E. coli* ESBL; the proportion was even higher amongst non-EU respondents (34.7%). Secondly, we found between 30 and 45% of all respondents neither followed the HICPAC nor the ESCMID recommendations requiring HCW to wear gowns and gloves at all times when entering the room of a patient in contact isolation [14]. In clinical practice it seems sufficient not to don a gown (and gloves) if no contact with blood or bodily fluid is anticipated, rendering more time urgently needed for care and treatment. In any case, the emphasis has to be on thorough education and proper implementation of standard precautions and hand hygiene as their integral component because they constitute the mainstay of controlling the spread of all micro-organisms (including MDROs).

Thirdly, contrary to these recommendations, between 10 and 20% of respondents from all countries did not consider any rooming specifications, e.g. cohorting or isolation for gram-positive MDRO. Up to 30% of all respondents abstained from such interventions in gram-negative MDRO, especially non-*E. coli* ESBL. These deficits seem somewhat alarming, since omitting such control measures is likely to facilitate the nosocomial spread of these organisms [15].

Our survey found the inability to separate patients colonized or infected with MDRO was due to the lack of personnel and insufficient single rooms, rather than a consequence of guideline scepticism or evidence-base paucity. Isolation practices implementation barriers were similar to those found for MRSA interventions in USA HCW interviews [16]. These findings underpin the view that the greatest challenge to implement contact precautions/isolation is the need for more staffing and isolation facilities, reinforced by a strong infection prevention ethos amongst HCWs and supported by a skilled infection control team as outlined in a previous European project [17].

A more recent survey among members of the Society for Healthcare Epidemiology of America (SHEA) on contact precaution use for MRSA and GRE revealed that over 60% of respondents were interested in alternative approaches, such as enhanced standard precautions and environmental cleaning/disinfection or targeted contact precautions and isolation (e.g., in conditions enhancing horizontal spread, such as diarrhoea or urinary incontinence) [18]. Our survey underlines that risk-stratified precautions are implemented for ESBL-E in few institutions or countries, respectively.

However, whether limiting contact precaution to those who have diarrhoea or urinary incontinence is equally effective in reducing transmission than application of contact precautions irrespective of the presence of risk factors, and whether this newer approach may be considered for gram-positive as well as gram-negative MDROs, remains to be determined in future studies and are matters of some urgency.

The strengths of this survey were its comprehensiveness about use of personal protective equipment and augmenting the response with on-site recruitment using a booth at ECCMID. Compared to other surveys we explicitly differentiated between *E. coli* and other Enterobacteriaceae, since the transmission risk of ESBL *E. coli* is deemed to be lower compared to non-*E. coli* ESBL, at least in the acute care setting [3, 19, 20]. The survey encompassed a broad geographical area across the world, including 32 EU and 28 non-EU countries.

Our study has some limitations. The online survey was potentially available to approx. 7000 ESCMID members and the ECCMID attendance was 10,839. Thus, the response rate was very poor, but still of significant size to draw interesting conclusions. Also, ECCMID attendants may have differed from other infection control experts and 10% of participants, though mostly non-clinicians with less experience in infection control and infectious diseases, showed unexpectedly poor knowledge about their local practice.

We therefore would urge some caution in generalising from these results, but they are a worrying potential indicator of variability in recommended practices, and are surely causes for concern which cannot be ignored. Larger studies, perhaps by individual countries, are required and measures to relieve recognised hindrances to improvement reflected upon and implemented.

The need for more rigorous studies comparing standard precautions to contact precautions/isolation in reducing the spread of MDRO has been previously highlighted [18]. These are essential to informing the best prevention strategies to combat spread of MDRO. The lack of uniform positive effects of contact isolation to prevent transmission may be explained by the variability of interpretation of this term. Indications for contact isolation require a global definition and further sound studies. ESCMID, HICPAC and any other MDR guidelines could perhaps add a score to the current infection control guidelines that would allow estimation of the level of implementation of contact precautions.

## Conclusion

We discovered great variation in components of the specific measures of contact precaution and isolation and mixed levels of implementation.

Our findings should inform the design of future trials ensuring that the methodology and different levels of contact precautions need to be described clearly to enhance comparability between studies.

## Additional files

Additional file 1: **Table S1.** Country of workplace of the 213 survey participants (number of respondents per country). **Table S2.** Survey respondents affiliations (*n* = 213). **Table S3.** MRSA contact precaution measures according to professional background. **Table S4.** GRE contact precaution measures according to professional background. **Table S5.** ESBL-*E. coli* contact precaution measures according to professional background. **Table S6.** ESBL-non-*E. coli* contact precaution measures according to professional background. **Table S7.** CR-*E. coli* contact precaution measures according to professional background. **Table S8.** CRE contact precaution measures according to professional background. **Table S9.** MRD *P. aeruginosa* contact precaution measures according to professional background. **Table S10.** MRD A. baumannii contact precaution measures according to professional background. **Table S11.** Indication and specification for contact precautions (CP) and isolation (cont. Next page) after deduplication*. **Table S12.** Other specific requirements for CP, results after deduplication*. **Table S13.** Characteristics of respondents that indicated “unknown” compared to respondents that provided any other answer. (DOCX 78 kb)

## Abbreviations

CRE: Carbapenem-resistant enterobacteriaceae
ECCMID: European congress on clinical microbiology and infectious diseases
ESBL: Extended-spectrum-beta-lactamase
ESCMID: European society of clinical microbiology and infectious diseases
ESGNI: ESCMID nosocomial infection study group
EU: European
GRE: Glycopeptide-resistant enterococci
HCW: Health-care workers
HICPAC: Healthcare infection control practices advisory committee
ICP: Infection control practitioner
IQR: Interquartile range
MDRO: Multidrug resistant organism
MRSA: Methicillin resistant *Staphylococcus aureus*
