# Supplementary material for: Variability in contact precautions to control the nosocomial spread of multi-drug resistant organisms in the endemic setting: a multinational cross-sectional survey
Source: Antimicrob Resist Infect Control. 2018 Jul 9;7:81. doi: 10.1186/s13756-018-0366-5 (PMC6038251; doi:10.1186/s13756-018-0366-5)
Supplement: Supplementary file 1 — Table S1. Country of workplace of the 213 survey participants (number of respondents per country). Table S2. Survey respondents affiliations (n = 213). Table S3. MRSA contact precaution measures according to professional background. Table S4. GRE contact precaution measures according to professional background. Table S5. ESBL-E. coli contact precaution measures according to professional background. Table S6. ESBL-non-E. coli contact precaution measures according to professional background. Table S7. CR-E. coli contact precaution measures according to professional background. Table S8. CRE contact precaution measures according to professional background. Table S9. MRD P. aeruginosa contact precaution measures according to professional background. Table S10. MRD A. baumannii contact precaution measures according to professional background. Table S11. Indication and specification for contact precautions (CP) and isolation (cont. Next page) after deduplication*. Table S12. Other specific requirements for CP, results after deduplication*. Table S13. Characteristics of respondents that indicated “unknown” compared to respondents that provided any other answer. (DOCX 78 kb) [file 13756_2018_366_MOESM1_ESM.docx]

**Additional file**

**Variability in contact precautions to control the nosocomial spread of multi-drug resistant organisms in the endemic setting: a multinational cross-sectional survey**

Danielle Vuichard Gysin^1,£^, Markus Dettenkofer^2^, Henri Saenz^3^, Barry Cookson^4^, and Andreas F. Widmer^1*^ for the ESCMID Study Group for Nosocomial Infections (ESGNI)

**Table S1: Country of workplace of the 213 survey participants (number of respondents per country).**

| **Northern Europe** | **Western Europe** | **Eastern Europe** | **Southern Europe *and* transcontinental Eurasian countries** |
| --- | --- | --- | --- |
| Denmark (4) | Austria (9) | Bulgaria (1) | Andorra (1) |
| Estonia (2) | Belgium (2) | Czech Republic (3) | Georgia (1) |
| Finland (1) | France (2) | Hungary (3) | Greece (6) |
| Ireland (3) | Germany (9) | Republic of Moldova (1) | Italy (11) |
| Lithuania (1) | Switzerland (9) | Poland (1) | Kosovo (1) |
| Norway (1) | The Netherlands (16) | Russian Federation (5) | Portugal (8) |
| Sweden (4) |  | Slovakia (1) | Romania (7) |
| United Kingdom (17) |  |  | Serbia (2) |
|  |  |  | Slovenia (5) |
|  |  |  | Spain (16) |
|  |  |  | Turkey (9) |
|  |  |  |  |
| **Africa** | **South America** | **Oceania** | **Asia and the Middle East** |
| Egypt (1) | Argentina (6) | Australia (1) | India (5) |
| Libya (2) | Brazil (3) |  | Islamic Republic of Iran (4) |
| Mauritius (1) | Chile (1) |  | Israel (2) |
| Nigeria (3) | Colombia (3) |  | Jordan (1) |
| South Africa (1) | Ecuador (2) |  | Kuwait (1) |
| Tunisia (2) | Peru (1) |  | Oman (2) |
|  |  |  | Republic of Korea (1) |
|  |  |  | Saudi Arabia (3) |
|  |  |  | United Arab Emirates (2) |
|  |  |  | Uzbekistan (1) |
|  |  |  | Thailand (2) |

**Table S2. Survey respondents affiliations (n=213)**

| - Infection Control and Prevention (ICP) | 20 (9.4%) |
| --- | --- |
| - ICP plus Microbiology and/or infectious diseases background | 57 (26.8%) |
| - Microbiology and/or ID (but not ICP) | 108 (50.7%) |
| - No speciality indicated | 20 (9.4%) |
| - other specialities (Veterinary medicine, Internal medicine, Pharmacy, Immunology, Lab technician in Microbiology, Manager Pharmacy) | 8 (3.8%) |

**Table S3. MRSA contact precaution measures according to professional background**

|  | **No CP** | **CP only if infected** | **CP if colonised and/or infected** | | **unknown** | **p-value** |
| --- | --- | --- | --- | --- | --- | --- |
| **ICP (n=72) vs.** | 5.6% | 8.3% | 86.1% | | 0.0% | **<0.001** |
| **non-ICP (n=127)** | 21.3% | 18.9% | 56.7% | | 3.1% |  |
| **Clinician (150) vs.** | 14.0% | 14.7% | 71.3% | | 0.0% | **0.002** |
| **non-clinician (49)** | 20.4% | 16.3% | 55.1% | | 8.2% |  |
|  | | | | | | |
|  | **Not determined or other** | **Gowns and gloves whenever entering the room** | | **Gowns and gloves if direct contact is anticipated** | | **p-value** |
| **ICP (n= 65) vs.** | 9.2% | 55.4% | | 35.4% | | 0.461 |
| **non-ICP (n=95)** | 4.2% | 58.9% | | 36.8% | |  |
| **Clinician (n=128) vs.** | 7.0% | 59.4% | | 33.6% | | 0.323 |
| **non-clinician (n=32)** | 3.1% | 50.0% | | 46.9% | |  |
|  | | | | | | |
|  | **no specific measures** | **Single room** | **Cohorting** | | **Spatial separation** | **p-value** |
| **ICP (n=68) vs.** | 4.4% | 69.1% | 14.7% | | 11.8% | 0.094 |
| **non-ICP (n=124)** | 16.1% | 59.7% | 16.1% | | 8.1% |  |
| **Clinician (n=148) vs.** | 11.50% | 62.20% | 16.90% | | 9.50% | 0.832 |
| **non-clinician (n=44)** | 13.60% | 65.90% | 11.40% | | 9.10% |  |

**Table S4. GRE contact precaution measures according to professional background**

|  | **No CP** | **if vancomycin resistant (VanA or VanB)** | **if teicoplanin resistant** | | **unknown** | **p-value** |
| --- | --- | --- | --- | --- | --- | --- |
| **ICP (n=72)** | 15.3% | 76.4% | 0.0% | | 8.3% | **0.031** |
| **non-ICP (n=127)** | 20.5% | 59.1% | 6.3% | | 14.2% |  |
| **Clinician (n=150)** | 16.70% | 76.00% | 0.70% | | 6.70% | **<0.001** |
| **non-clinician (n=49)** | 24.50% | 46.90% | 0.0% | | 28.60% |  |
|  | **Not determined or other** | **Gowns and gloves whenever entering the room** | | **Gowns and gloves if direct contact is anticipated** | | **p-value** |
| **ICP (n=54)** | 11.1% | 51.9% | | 37.0% | | **0.007** |
| **non-ICP (n=81)** | 0.0% | 63.0% | | 37.0% | |  |
| **Clinician (n=112)** | 5.4% | 59.8% | | 34.8% | | 0.318 |
| **non-clinician (n=23)** | 0.0% | 52.2% | | 47.8% | |  |
|  | **no specific measures** | **Single room** | **Cohorting** | | **Spatial separation** | **p-value** |
| **ICP (n=68)** | 10.3% | 67.6% | 14.7% | | 7.4% | 0.254 |
| **non-ICP (n=123)** | 19.5% | 53.7% | 17.1% | | 9.8% |  |
| **Clinician (n= 147)** | 13.6% | 63.9% | 15.6% | | 6.8% | **0.028** |
| **non-clinician (n=44)** | 25.0% | 40.9% | 18.2% | | 15.9% |  |

**Table S5. ESBL-*E. coli* contact precaution measures according to professional background**

|  | **No CP** | **only if infected** | **if colonised and/or infected** | | **ESBL not determined** | | **unknown** | **p-value** |
| --- | --- | --- | --- | --- | --- | --- | --- | --- |
| **ICP (n=72)** | **34.7%** | **11.1%** | **50.0%** | | **0.0%** | | **4.20%** | 0.220 |
| **non-ICP (n=127)** | **32.3%** | 15**.0%** | **40.2%** | | **4.7%** | | **7.90%** |  |
| **Clinician (n=150)** | 35.30% | 12.00% | 48.00% | | 1.30% | | 3.30% | **0.001** |
| **non-clinician (n=49)** | 26.50% | 18.40% | 30.60% | | 8.20% | | 16.30% |  |
|  | **Not determined or other** | **Gowns and gloves whenever entering the room** | | | **Gowns and gloves if direct contact is anticipated** | | | **p-value** |
| **ICP (n=42)** | 2.4% | 47.6% | | | 50.0% | | | 0.582 |
| **non-ICP (n=69)** | 0.0% | 47.8% | | | 52.2% | | |  |
| **Clinician (n=87)** | 1.1% | 47.1% | | | 51.7% | | | 0.852 |
| **non-clinician (n=24)** | 0.0% | 50.0% | | | 50.0% | | |  |
|  | **no specific measures** | **Single room** | | **Cohorting** | | **Spatial separation** | | **p-value** |
| **ICP (n=68)** | 36.8% | 32.4% | | 13.2% | | 17.6% | | 0.426 |
| **non-ICP (n=122)** | 33.6% | 32.0% | | 22.1% | | 12.3% | |  |
| **Clinician (n=147)** | 36.1% | 34.0% | | 19.0% | | 10.9% | | 0.106 |
| **non-clinician (n=43)** | 30.2% | 25.6% | | 18.6% | | 25.6% | |  |

**Table S6. ESBL-non-*E. coli* contact precaution measures according to professional background**

|  | **No CP** | **only if infected** | **if colonised and/or infected** | **ESBL not determined** | **unknown** | **p-value** |
| --- | --- | --- | --- | --- | --- | --- |
| **ICP (n=72)** | 25.0% | 11.1% | 56.9% | 0.0% | 6.90% | 0.190 |
| **non-ICP (n=127)** | 26.8% | 16.5% | 43.3% | 4.7% | 8.70% |  |
| **Clinician (n=150)** | 26.70% | 15.30% | 52.70% | 1.30% | 4.00% | **<0.001** |
| **non-clinician (n=49)** | 24.50% | 12.20% | 34.70% | 8.20% | 20.40% |  |
|  | **Not determined or other** | **Gowns and gloves whenever entering the room** | | **Gowns and gloves if direct contact is anticipated** | | **p-value** |
| **ICP (n=49)** | 12.2% | 44.9% | | 42.9% | | 0.125 |
| **non-ICP (n=73)** | 2.7% | 50.7% | | 46.6% | |  |
| **Clinician (n=99)** | 8.10% | 47.50% | | 44.40% | | 0.370 |
| **non-clinician (n=23)** | 0.00% | 52.20% | | 47.80% | |  |
|  | **no specific measures** | **Single room** | **Cohorting** | **Spatial separation** | | **p-value** |
| **ICP (n=68)** | 27.9% | 39.7% | 14.7% | 17.6% | | 0.428 |
| **non-ICP (n=122)** | 31.1% | 32.0% | 23.0% | 13.9% | |  |
| **Clinician (n=147)** | 31.3% | 36.7% | 21.1% | 10.9% | | **0.022** |
| **non-clinician (n=43)** | 25.6% | 27.9% | 16.3% | 30.2% | |  |

**Table S7. CR-*E. coli* contact precaution measures according to professional background**

|  | **No CP** | **only if infected** | **if colonised and/or infected** | | **unknown** | **p-value** |
| --- | --- | --- | --- | --- | --- | --- |
| **ICP (n=72)** | 14.2% | 15.0% | 57.5% | | 13.4% | **0.017** |
| **non-ICP (n=127)** | 9.7% | 9.7% | 77.8% | | 2.8% |  |
| **Clinician (n=150)** | 10.00% | 11.30% | 74.00% | | 4.70% | **<0.001** |
| **non-clinician (n=49)** | 20.40% | 18.40% | 36.70% | | 24.50% |  |
|  | **Not determined or other** | **Gowns and gloves whenever entering the room** | | **Gowns and gloves if direct contact is anticipated** | | **p-value** |
| **ICP (n=62)** | 6.5% | 58.1% | | 35.5% | | 0.466 |
| **non-ICP (n=87)** | 2.3% | 62.1% | | 35.6% | |  |
| **Clinician (n=123)** | 4.1% | 63.4% | | 32.5% | | 0.234 |
| **non-clinician (n=26)** | 3.8% | 46.2% | | 50.0% | |  |
|  | **no specific measures** | **Single room** | **Cohorting** | | **Spatial separation** | **p-value** |
| **ICP (n=68)** | 11.8% | 67.6% | 11.8% | | 8.8% | 0.280 |
| **non-ICP (n=122)** | 17.2% | 53.3% | 14.8% | | 14.8% |  |
| **Clinician (n=147)** | 11.6% | 63.9% | 14.3% | | 10.2% | **0.006** |
| **non-clinician (n=43)** | 27.9% | 39.5% | 11.6% | | 20.9% |  |

**Table S8. CRE contact precaution measures according to professional background**

|  | **No CP** | **CP only if infected** | **CP if colonised and/or infected** | **other (e.g. standard precautions)** | **unknown** | **p-value** |
| --- | --- | --- | --- | --- | --- | --- |
| **ICP (n=72)** | 11.1% | 6.9% | 79.2% | 0.0% | 2.8% | 0.059 |
| **non-ICP (n=127)** | 10.2% | 15.7% | 63.8% | 2.4% | 7.9% |  |
| **Clinician (n=150)** | 8.7% | 12.0% | 76.0% | 0.7% | 2.7% | **<0.001** |
| **non-clinician (n=49)** | 16.3% | 14.3% | 49.0% | 4.1% | 16.3% |  |
|  | **Not determined or other** | **Gowns and gloves whenever entering the room** | | **Gowns and gloves if direct contact is anticipated** | | **p-value** |
| **ICP (n=59)** | 6.8% | 61.0% | | 32.2% | | 0.528 |
| **non-ICP (n=96)** | 3.1% | 59.4% | | 37.5% | |  |
| **Clinician (n=126)** | 4.8% | 62.7% | | 32.5% | | 0.279 |
| **non-clinician (n=29)** | 3.4% | 48.3% | | 48.3% | |  |
|  | **no specific measures** | **Single room** | **Cohorting** | **Spatial separation** | | **p-value** |
| **ICP (n=67)** | 10.4% | 73.1% | 11.9% | 4.5% | | 0.31 |
| **non-ICP (n=122)** | 12.3% | 61.5% | 14.8% | 11.5% | |  |
| **Clinician (n=147)** | 8.2% | 70.1% | 15.0% | 6.8% | | **0.004** |
| **non-clinician (n=42)** | 23.8% | 50.0% | 9.5% | 16.7% | |  |

**Table S9. MRD *P. aeruginosa* contact precaution measures according to professional background**

|  | **No CP** | **CP only if infected** | **CP if colonised and/or infected** | | **unknown** | **p-value** |
| --- | --- | --- | --- | --- | --- | --- |
| **ICP (n=72)** | 13.9% | 9.7% | 70.8% | | 5.6% | **0.077** |
| **non-ICP (n=127)** | 15.7% | 20.5% | 53.5% | | 10.2% |  |
| **Clinician (n=150)** | 15.3% | 14.0% | 66.0% | | 4.7% | **0.001** |
| **non-clincian (n=49)** | 14.3% | 24.5% | 40.8% | | 20.4% |  |
|  | **Not determined or other** | **Gowns and gloves whenever entering the room** | | **Gowns and gloves if direct contact is anticipated** | | **p-value** |
| **ICP (n=57)** | 12.3% | 43.9% | | 43.9% | | **0.035** |
| **non-ICP (n=90)** | 3.3% | 61.1% | | 35.6% | |  |
| **Clinician (n=116)** | 7.8% | 53.4% | | 38.8% | | 0.659 |
| **non-clincian (n=31)** | 3.2% | 58.1% | | 38.7% | |  |
|  | **no specific measures** | **Single room** | **Cohorting** | | **Spatial separation** | **p-value** |
| **ICP (n=65)** | **13.8%** | **55.4%** | **13.8%** | | **16.9%** | **0.693** |
| **non-ICP (n=120)** | **15.8%** | **52.5%** | **19.2%** | | **12.5%** |  |
| **Clinician (n=143)** | **12.6%** | **56.6%** | **18.9%** | | **11.9%** | **0.076** |
| **non-clincian (n=42)** | 23.8% | 42.9% | 11.9% | | 21.4% |  |

**Table S10. MRD *A. baumannii* contact precaution measures according to professional background**

|  | **No CP** | **CP only if infected** | **CP if colonised and/or infected** | | **unknown** | **p-value** |
| --- | --- | --- | --- | --- | --- | --- |
| **ICP (n=72)** | 13.9% | 13.9% | 68.1% | | 4.2% | 0.086 |
| **non-ICP (n=127)** | 11.0% | 20.5% | 55.1% | | 13.4% |  |
| **Clinician (n=150)** | 10.7% | 17.3% | 68.0% | | 4.0% | **<0.001** |
| **non-clincian (n=49)** | 16.3% | 20.4% | 34.7% | | 28.6% |  |
|  | **Not determined or other** | **Gowns and gloves whenever entering the room** | | **Gowns and gloves if direct contact is anticipated** | | **p-value** |
| **ICP (n=50)** |  | 56.0% | | 44.0% | | 0.593 |
| **non-ICP (n=90)** |  | 61.1% | | 38.9% | |  |
| **Clinician (n=114)** |  | 61.4% | | 38.6% | | 0.286 |
| **non-clincian (n=26)** |  | 50.0% | | 50.0% | |  |
|  | **no specific measures** | **Single room** | **Cohorting** | | **Spatial separation** | **p-value** |
| **ICP (n=67)** | 13.4% | 62.7% | 16.4% | | 7.5% | 0.608 |
| **non-ICP (n=121)** | 14.0% | 54.5% | 18.2% | | 13.2% |  |
| **Clinician (n=146)** | 11.0% | 63.7% | 17.1% | | 8.2% | **0.004** |
| **non-clincian (n=42)** | 23.8% | 35.7% | 19.0% | | 21.4% |  |

**Table S11. Indication and specification for contact precautions (CP) and isolation (cont. next page) after deduplication***

|  | **MRSA^§^** | | | **VRE^§^** | | | ***E. coli* ESBL^§^** | | | **Non-*E. coli* ESBL^§^** | | |
| --- | --- | --- | --- | --- | --- | --- | --- | --- | --- | --- | --- | --- |
|  | **EU** | **Non EU** | **p-value** | **EU** | **Non EU** | **p-value** | **EU** | **Non EU** | **p-value** | **EU** | **Non EU** | **p-value** |
| **No CP** | 10 (11.1) | 8 (19.5) | 0.429 | 16 (19.8) | 8 (22.2) | 0.346 | 27 (30.0) | 12 (29.3) | 0.430 | 18 (20.0) | 12 (29.3) | **0.046** |
| **CP only if infected** | 15 (16.7) | 5 (12.2) |  | 14 (17.3) | 10 (27.8) |  | 18 (20.0) | 4 (9.8) |  | 20 (22.2) | 3 (7.3) |  |
| **CP if colonised and/or infected** | 62 (68.9) | 28 (68.3) |  | 51 (63.0) | 18 (50.0) |  | 37 (41.1) | 18 (43.9) |  | 44 (48.9) | 17 (41.5) |  |
| **Unknown** | 3 (3.3) | 0 |  | 0 | 0 |  | 5 (5.6) | 5 (12.2) |  | 5 (5.6) | 7 (17.1) |  |
| **ESBL not determined** | n.a. | n.a. |  | n.a. | n.a. |  | 3 (3.3) | 2 (4.9) |  | 3 (3.3) | 2 (4.9) |  |
| **Total respondents** | 90 (68.7) | 41 (31.3) |  | 67 (72.8) | 25 (27.2) |  | 90 (68.7) | 41 (31.3) |  | 90 (68.7) | 41 (31.3) |  |
| **Gowns and gloves whenever entering the room** | 36 (48.0) | 18 (60.0) | 0.406 | 37 (55.2) | 13 (52.0) | 0.901 | 18 (32.1) | 12 (63.2) | **0.017** | 20 (34.5) | 13 (72.2) | **0.005** |
| **Gowns and gloves if direct contact is anticipated** | 35 (46.7) | 12 (40.0) |  | 28 (41,8) | 12 (48.0) |  | 38 (67.9) | 7 (36.8) |  | 38 (65.5) | 5 (27.8) |  |
| **Other procedures (e.g. standard precautions only)** | 4 (5.3) | 0 (0.0) |  | 2 (3.0) | 0 (0.0) |  | 0 (0.0) | 0 (0.0) |  | 0 (0.0) | 0 (0.0) |  |
| **Total respondents** | 75 (71.4) | 30 (28.6) |  | 67 (72.8) | 25 (27.2) |  | 56 (74.7) | 19 (25.3) |  | 58 (76.3) | 18 (23.7) |  |
| **Single room** | 53 (61.6) | 26 (66.7) | 0.235 | 48 (61.5) | 21 (58.3) | 0.556 | 23 (27.2) | 14 (35.0) | 0.682 | 27 (32.5) | 12 (30.0) | 0.434 |
| **Cohorting** | 14 (16.3) | 4 (10.3) |  | 12 (15.4) | 5 (13.9) |  | 17 (20.5) | 7 (17.5) |  | 19 (22.9) | 7 (17.5) |  |
| **Spatial separation^¥^** | 9 (10.5) | 3 (7.7) |  | 8 (10.3) | 2 (5.6) |  | 13 (15.7) | 8 (20.0) |  | 11 (13.3) | 10 (25.0) |  |
| **No specific measures** | 10 (11.6) | 6 (15.4) |  | 10 (12.8) | 8 (22.2) |  | 30 (36.1) | 11 (27.5) |  | 26 (31.3) | 11 (27.5) |  |
| **Total respondents** | 86 (68.8) | 39 (31.2) |  | 78 (68.4) | 36 (31.6) |  | 83 (67.5) | 40 (32.5) |  | 83 (67.5) | 40 (32.5) |  |

* Criteria for duplicate: same country and same hospital size

**^§^** Values are indicated as absolute numbers and percentages (%) are related to the corresponding total respondents

^¥^ Shared room with MDRO-negative patients but with optical barrier (e.g. red margin on the floor) or separated by screen/curtains

|  | **Carbapenem resistant *E. coli*** | | | **Carbapenem resistant non-*E. coli*** | | | **MDR *P. aeruginosa*** | | | **MDR *A. baumannii*** | | |
| --- | --- | --- | --- | --- | --- | --- | --- | --- | --- | --- | --- | --- |
|  | **EU** | **Non EU** | **p-value** | **EU** | **Non EU** | **p-value** | **EU** | **Non EU** | **p-value** | **EU** | **Non EU** | **p-value** |
| **No CP** | 11 (12.2) | 6 (14.6) | 0.890 | 8 (9.0) | 7 (17.1) | 0.222 | 12 (13.3) | 7 (17.1) | 0.795 | 7 (7.8) | 7 (17.1) | 0.432 |
| **CP only if infected** | 15 (16.7) | 5 (12.2) |  | 15 (16.9) | 4 (9.8) |  | 18 (20.0) | 6 (14.6) |  | 18 (20.0) | 9 (22.0) |  |
| **CP if colonised and/or infected** | 57 (63.3) | 26 (63.4) |  | 61 (68.5) | 27 (65.9) |  | 51 (56.7) | 25 (61.0) |  | 55 (61.1) | 21 (51.2) |  |
| **Unknown** | 7 (7.8) | 4 (9.8) |  | 5 (5.6) | 3 (7.3) |  | 9 (10.0) | 3 (7.3) |  | 10 (11.1) | 4 (9.8) |  |
| **Total respondents** | 90 (68.7) | 41 (31.3) |  | 89 (68.5) | 41 (31.5) |  | 90 (68.7) | 41 (31.3) |  | 90(68.7) | 41 (31.3) |  |
| **Gowns and gloves whenever entering the room** | 36 (52.9) | 18 (66.7) | 0.223 | 39 (55.7) | 17 (63.0) | 0.517 | 29 (46.8) | 19 (65.5) | 0.095 | 34 (50.7) | 16 (61.5) | 0.349 |
| **Gowns and gloves if direct contact is anticipated** | 32 (47.1) | 9 (33.3) |  | 31 (44.3) | 10 (37.0) |  | 33 (53.2) | 10 (34.5) |  | 33 (49.3) | 10 (23.3) |  |
| **Other procedures** | 0 | 0 |  | 0 | 0 |  | 0 | 0 |  | 0 | 0 |  |
| **Total respondents** | 68 (71.6) | 27 (28.4) |  | 70 (72.2) | 27 (27.8) |  | 62 (68.1) | 29 (31.9) |  | 67 (72.0) | 26 (28.0) |  |
| **Single room** | 48 (57.1) | 19 (47.5) | 0.506 | 57 (68.7) | 21 (52.5) | 0.222 | 45 (55.6) | 20 (50.0) | 0.206 | 49 (59.0) | 21 (52.5) | 0.272 |
| **Cohorting** | 11 (13.1) | 4 (10.0) |  | 11 (13.3) | 6 (15.0) |  | 17 (21.0) | 4 (10.0) |  | 15 (18.1) | 4 (10.0) |  |
| **Spatial separation^¥^** | 11 (13.1) | 9 (22.5) |  | 6 (7.2) | 7 (17.5) |  | 10 (12.3) | 8 (20.0) |  | 8 (9.6) | 8 (20.0) |  |
| **No specific measures** | 14 (16.7) | 8 (20.0) |  | 9 (10.8) | 6 (15.0) |  | 9 (11.1) | 8 (20.0) |  | 11 (13.3) | 7 (17.5) |  |
| **Total respondents** | 84 (67.7) | 40 (32.3) |  | 83 (67.5) | 40 (32.5) |  | 81 (66.9) | 40 (33.1) |  |  |  |  |

**Table S12. Other specific requirements for CP, results after deduplication*:**

| **MDRO** | **Origin of responses** | **Total responses** | **Additional pre-emptive CP based on patient’s history^Θ^** | **CP only required specific risk factors present**^£^ | **Additional pre-emptive CP but only if specific risk factors** | **None applicable** | **p-value** |
| --- | --- | --- | --- | --- | --- | --- | --- |
| **MRSA** | **EU** | 77 | 70 (90.9) | 1 (1.3) | 1 (1.3) | 5 (6.5) | **0.156** |
|  | **Non EU** | 26 | 26 (78.8) | 0 (0.0) | 2 (6.1) | 5 (15.2) |  |
| **GRE** | **EU** | 59 | 50 (84.7) | 2 (3.4) | 1 (1.7) | 6 (10.2) | 0.259 |
|  | **Non EU** | 24 | 19 (79.2) | 2 (8.3) | 2 (8.3) | 1 (4.2) |  |
| **ESBL *E. coli*** | **EU** | 57 | 43 (75.4) | 8 (14.0) | 3 (5.3) | 3 (5.3) | 0.629 |
|  | **Non EU** | 23 | 19 (82.6) | 1 (4.3) | 2 (8.7) | 1 (4.3) |  |
| **ESBL non-*E. coli*** | **EU** | 63 | 51 (81.0) | 5 (7.9) | 3 (4.8) | 4 (6.3) | 0.131 |
|  | **Non EU** | 20 | 15 (75.0) | 3 (15.0) | 2 (10.0) | 0 (0.0) |  |
| **Carbapenem resistant *E. coli*** | **EU** | 73 | 59 (80.8) | 3 (4.1) | 4 (5.5) | 7 (9.6) | 0.631 |
|  | **Non EU** | 31 | 28 (90.3) | 0 (0.0) | 2 (6.5) | 1 (3.2) |  |
| **Carbapenem resistant Enterobacteriaceae (non-*E. coli*)** | **EU** | 76 | 65 (85.5) | 2 (2.6) | 4 (5.3) | 5 (6.6) | 0.952 |
|  | **Non EU** | 31 | 27 (87.1) | 1 (3.2) | 4 (5.3) | 2 (6.5) |  |
| **MDR *P. aeruginosa*** | **EU** | 65 | 53 (81.5) | 3 (4.6) | 4 (6.2) | 5 (7.7) | 0.271 |
|  | **Non EU** | 30 | 27 (90.0) | 0 (0.0) | 3 (10.0) | 0 (0.0) |  |
| **MDR A. baumannii** | **EU** | 71 | 62 (87.3) | 1 (1.4) | 5 (7.0) | 3 (4.2) | 1.000 |
|  | **Non EU** | 29 | 26 (89.7) | 0 (0.0) | 2 (6.9) | 1 (3.4) |  |

* Criteria for duplication: same country and same hospital size

**^Θ^** Confirmation by culture not required (e.g. formerly positive for respective MDRO or presumptive infection/colonization with respective MDRO)

^£^ CP only when certain risk factors present such as incontinence, diarrhoea, poor personal hygiene (e.g. due to mental disorder)

**Table S13. Characteristics of respondents that indicated “unknown” compared to respondents that provided any other answer.**

|  | **CP measures for MRSA** | | | **CP measures for GRE** | | |
| --- | --- | --- | --- | --- | --- | --- |
|  | **Unknown (n=4)** | **Any other answer (n=195)** | **p-value*** | **unknown (n=24)** | **Any other answer (n=175)** | **p-value*** |
| **MD as profession** | 0 | 150 | **0.003** | 10 | 140 | **<0.001** |
| **Working in hospital** | 1 | 157 | **0.028** | 11 | 161 | **<0.001** |
| **Working in infectious diseases (ID), Microbiology and/or infection control** | 2 | 175 | 0.061 | 17 | 147 | 0.148 |
| **average years of experience in infection control (years)** | 1.25 | 10.2 | **0.041** | 6 | 10.6 | **0.014** |
|  | | | | | | |
|  | **CP measures ESBL E. Coli** | | | **CP measures for non-*E. coli* ESBL** | | |
|  | **Unknown (n=13)** | **Any other answer (n=180)** | **p-value*** | **Unknown (n= 16)** | **Any other answer (n= 177)** | **p-value*** |
| **MD as profession** | 5 | 143 | 0.003 | 6 | 142 | 0.001 |
| **Working in hospital** | 7 | 164 | 0.001 | 8 | 148 | 0.004 |
| **Working in ID, Microbiology and/or infection control** | 10 | 149 | 0.704 | 13 | 161 | 0.375 |
| **average years of experience in infection control (years)** | 5.4 | 10.5 | **0.040** | 6.8 | 10.5 | 0.108 |

* Fisher's exact test or student t-test as appropriate, 2-tailed (missing excluded on a case by case basis)
